# Relative overexpression of membrane-bound versus soluble TNF in spondyloarthritis

**DOI:** 10.1186/1479-5876-10-S3-P34

**Published:** 2012-11-28

**Authors:** Carmen A Ambarus, Leonie M van Duivenvoorde, Huriatul Masdar, Melissa N van Tok, Paul-Peter Tak, Nataliya G Yeremenko, Dominique L Baeten

**Affiliations:** 1 Dept. of Clinical Immunology and Rheumatology, Academic Medical Center, Amsterdam, the Netherlands

## Background

Macrophages and their pro-inflammatory cytokines, including TNF, are pivotal mediators of chronic synovitis in Rheumatoid Arthritis (RA) and Spondyloarthritis (SpA). Despite similar levels of synovial macrophage infiltration and clinical responses to TNF-blockade, SpA is characterized by more pronounced infiltration with alternatively activated macrophages and ongoing osteoproliferation. Here, we investigated whether these differences were related to differential expression and/or function of TNF between both diseases.

## Methods

Expression of membrane-bound TNF (mTNF) and soluble TNF (sTNF) was measured in IFN-γ, IL-4 or IL-10 polarized macrophages obtained from healthy donors. Expression of TNF and its receptors was also measured in synovial fluid (SF) and synovial tissue (ST) of actively inflamed knee joints. Mice transgenically overexpressing mTNF were evaluated for spondylitis and arthritis.

## Results

The expression of mTNF was increased in IL-4 and IL-10 polarized macrophages compared to IFN-γ polarized macrophages. Moreover, secretion of sTNF was impaired in both alternatively polarized macrophages, indicating a shift from sTNF to mTNF. In line with these data, the sTNF SF levels were lower in SpA compared to RA (p=0.01) despite similar TNF mRNA levels in ST. This was not related to altered expression of TNF receptors as both TNF-R1 and TNF-R2 were similarly expressed in ST. mRNA levels of TACE, the enzyme responsible for the cleavage of TNF, were also similar between SpA and RA ST. To investigate the relevance of overexpressed mTNF in SpA pathophysiology, we characterized mTNF transgenic mice. As previously described, these mice develop a moderate arthritis, resulting in deformation of paws and loss of grip. Histological, the joints were characterized by synovitis, lymphoid aggregates and osteoproliferation. Besides arthritis, all mice spontaneously developed spondylitis as evidenced by a crinkled tail, a hunchback and histological pathophysiology.

## Conclusions

Membrane-bound TNF is relatively overexpressed by alternatively polarized macrophages in SpA synovitis and leads to an axial and peripheral SpA phenotype in transgenic mice.

